# Establishment of prediction equations for subcutaneous tissue thickness in two representative intramuscular deltoid injections

**DOI:** 10.1016/j.jvacx.2023.100316

**Published:** 2023-05-22

**Authors:** Ryosuke Kowatari, Hanae Sasaki, Kenyu Murata, Ken Sato, Koichi Sagawa, Masako Kudo, Masahito Minakawa

**Affiliations:** a Department of Thoracic and Cardiovascular Surgery, Hirosaki University School of Medicine, Japan; b Hirosaki University Health Administration Center, Japan; c Hirosaki University Graduate School of Science and Technology, Japan; d Hirosaki University Hospital Nursing Department, Japan

**Keywords:** Intramuscular injection, Vaccination, COVID-19, Shoulder injuries related to vaccine administratio

## Abstract

This study clarifies the predicted subcutaneous shoulder depth and investigates the safety of the conventional (three-finger breadth method) and new (axillary method) intramuscular injection methods. The anatomical features of 245 volunteers who received the COVID-19 vaccination via the conventional method were investigated at the injection site (T point) and the hypothetical injection site using the new method (A point) via ultrasonography. The body mass index (BMI) and subcutaneous thickness at the T point (men: r = 0.75; women: r = 0.45) and the A point (men: r = 0.81; women: r = 0.55) were positively correlated. The upper arm circumference and subcutaneous thickness at the T point (r = 0.51) and the A point (r = 0.58) were correlated in women. Formulas to predict subcutaneous thickness using BMI and upper arm circumference were established: predicted subcutaneous thickness at the A point = 0.62 × BMI − 7.7 mm (R^2^ = 0.66) in men and 0.658 × BMI − 5.5 mm (R^2^ = 0.31) in women. This study demonstrates safe intramuscular injection sites and their depth.

## Introduction

Intramuscular injection is a popular method of vaccine administration, and the deltoid muscle is the preferred injection site in adults. Shoulder injuries related to vaccine administration (SIRVA) are well-known complications that should be avoided. SIRVA are defined by the National Vaccine Injury Compensation Program as “shoulder pain with limited range of motion within 48 h after vaccine reception in individuals with no prior history of pain, inflammation, or dysfunction of the affected shoulder before vaccine administration” [1].

In recent years, the COVID-19 pandemic and increased vaccinations have led to several opportunities for intramuscular injections [2], and a rapid increase in vaccine administration has resulted in an increased risk and incidence of SIRVA [2], [3], [4], [5], [6]. Therefore, it is becoming increasingly important to consider appropriate intramuscular injection methods.

As the injection technique can be adjusted to prevent SIRVA, several studies regarding the proper technique have been conducted, resulting in several suggestions for appropriate injection sites and depths [7], [8] However, few studies have re-examined the validity of those previous studies. The purpose of this study was to investigate the correlations between body mass index (BMI) and upper arm circumference with subcutaneous thickness and to establish formulas for predicting the subcutaneous thickness for safe intramuscular injection. Furthermore, the safety of the conventional intramuscular method and the newly recommended method were examined.

## Materials and methods

### Study design

This research was conducted in accordance with the Helsinki Declaration. This study was conducted at Hirosaki University Hospital during the Hirosaki University 3rd COVID-19 Vaccine Mass Inoculation in 2022 from February 9 to March 27, 2022. The institutional board of Hirosaki University approved this study (approval number 2021–140). Written informed consent was obtained from all participants.

### Participants

Of 5,884 individuals vaccinated during the study period, 245 (134 men and 111 women) were included in this study. The volunteers included university students and employees, excluding medical staff. All volunteers provided written, informed consent for their participation in the study and for its publication. The participants received the mRNA COVID-19 vaccine; 31 participants received the Pfizer-BioNTech vaccine and 214 received the Moderna vaccine. The injection site was standardized to three finger breadths (approximately 5 cm) below the mid-acromion [9], which is frequently used in Japan.

### Data collection

**Fig. 1:**
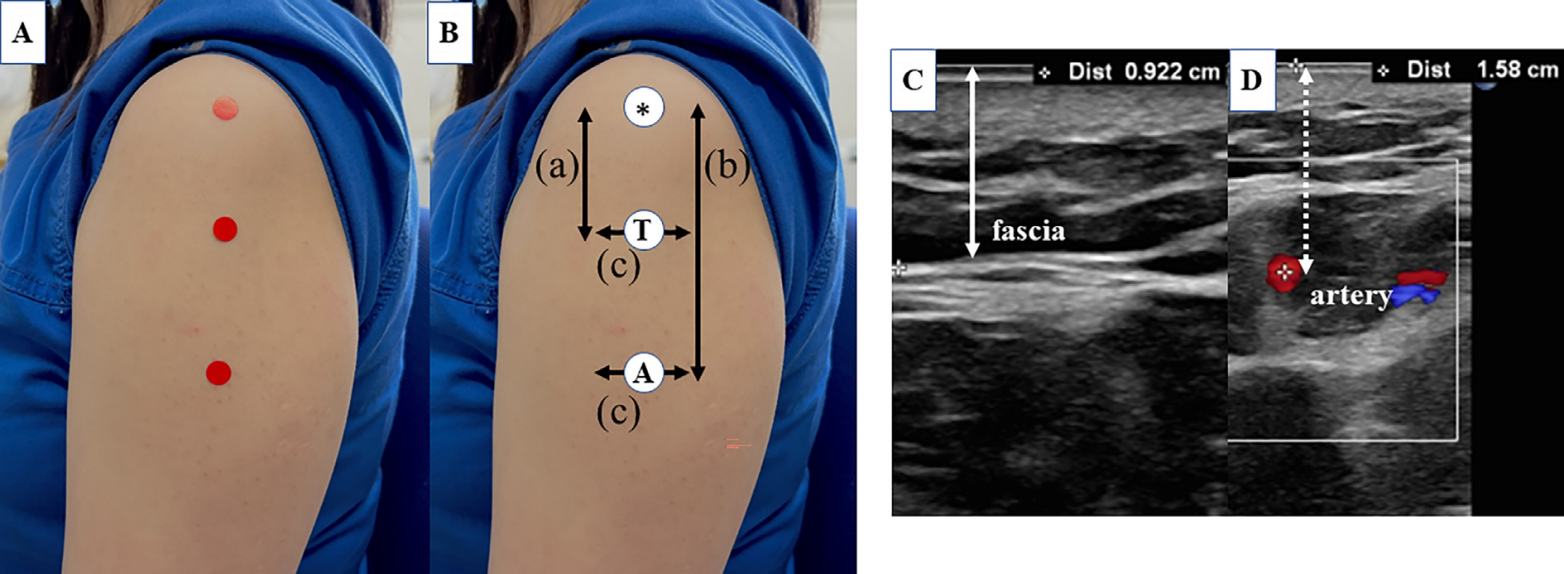
**(A, B)** The points that were investigated in this study are shown. * indicates the midportion of the mid-acromion lateral border. T represents the point at which the vaccine injection was performed using the three-finger breadth method. A represents the point of vaccine injection using the axillary method. (a) represents the length between * and the T point. (b) represents the length between * and the A point. (c) represents the length between the adjacent artery visible on ultrasound and the T or A point. **(C, D)** Ultrasound images of a male volunteer show the distance between the skin to the fascia at deltoid muscle (arrow) and the distance between the skin to the adjacent artery (dotted arrow) at the T point.

The locations of the examined sites are shown in Fig. 1. Two sites in the deltoid muscle were examined: the T point, a site three fingers below the acromion, which is frequently used in Japanese clinical practice, and the A point, the intersection of a vertical line from the axillary line and a horizontal line connecting the upper edge of the anterior axillary line and the upper edge of the posterior axillary line, which was reported by Nakajima et al. in 2017 as the appropriate intramuscular injection site [7].

The participants’ heights and weights were recorded, and the BMIs were calculated. The acromion was marked prior to vaccination. The vaccine was injected intramuscularly at the T point by three trained nurses using 15-mm long 25 G needles. After vaccination, the injection site was marked (T point). Once hemostasis is achieved by compression of the injection site, the A point was determined by a physician using ultrasound. The distances from the acromion to the T point and the A point were measured. The circumference of the arm was measured at the level of the A point.

The depths from the skin to the deltoid fascia (the subcutaneous thickness) and to the adjacent artery (the posterior circumflex humeral artery or its branches) at the T point and the A point were measured using a linear-array high-frequency probe (Sonosite Edge, Fujifilm, Tokyo, Japan) held perpendicularly to the test site. Ultrasound images obtained in B-mode were used in this study, and doppler mode was used to detect the arteries. The arteries were quantitatively measured to determine their anterior or posterior displacement (Fig. 1). Data collection was performed by two doctors. The participants were monitored for complications like allergy, bleeding, and other symptoms of SIRVA for 15 min after receiving the vaccination.

### Statistical analysis

All statistical analyses were conducting using EZR (Saitama Medical Center, Jichi Medical University, Saitama, Japan). Statistical significance was set at p < 0.05. Separate analyses were performed for each sex. Pearson’s correlation analysis was performed to assess the strength of the relationships between the BMI and each depth (subcutaneous and skin to artery) at the T point and the A point and the circumference of the arm and each depth at the T point and the A point. A single linear regression was calculated to predict each depth at the T and the A point based on the BMI and each depth at the T point and the A point based on the circumference of the arm.

## Results

The mean age, BMI, and arm circumference of the male volunteers were 22.4 ± 4.6 years, 22.1 ± 3.3 kg/m^2^, and 299 ± 34 mm, respectively (Table 1). The mean age, BMI, and arm circumference of the female volunteers were 21.9 ± 4.6 years, 20.6 ± 1.8 kg/m^2^, and 270 ± 22 mm, respectively. The mean distance from the anterior edge of the mid-acromion lateral border to the T point was 49.5 ± 4.9 mm in men and 48.1 ± 5.6 mm in women. The mean distance from the anterior edge of the mid-acromion lateral border to the A point was 90.5 ± 13.8 mm in men and 81.5 ± 12.7 mm in women. The subcutaneous thickness at the T point was 4.7 ± 1.7 mm in men and 6.2 ± 1.7 mm in women. The subcutaneous thickness at the A point was 5.9 ± 2.5 mm in men and 8.0 ± 2.2 mm in women. The depth from the skin to the adjacent artery at the T point was 16.8 ± 4.3 mm in men and 16.5 ± 4.3 mm in women. At the A point, the depth from the skin to the adjacent artery was 18.3 ± 4.0 mm in men and 18.8 ± 4.1 mm in women. The artery was 4.2 ± 13.6 mm posterior to the T point in men and 2.5 ± 13.4 mm posterior to the T point in women. The artery was 2.5 ± 13.6 mm posterior to the A point in men and 3.7 ± 15.8 mm posterior to the A point in women. At the T point, the depth from the fascia to the adjacent artery was 12.0 ± 4.2 mm in men and 10.2 ± 4.4 mm in women. At the A point, the depth from the fascia to the adjacent artery was 12.2 ± 4.4 mm in men and 10.4 ± 4.7 mm in women.

**Table 1:** Participant characteristics.

		**Men (n = 134)**	**Women (n = 111)**
Age (years)		22.4 ± 4.6	21.9 ± 4.6
BMI (kg/m^2^)		22.1 ± 3.3	20.6 ± 1.8
Arm circumference (mm)		299 ± 34	270 ± 22
Acromion-the T point (mm)		49.5 ± 4.9	48.1 ± 5.6
Acromion- the A point (mm)		90.5 ± 13.8	81.5 ± 12.7
(T)	Subcutaneous thickness (mm)	4.7 ± 1.7	6.2 ± 1.7
	Depth from skin to artery (mm)	16.8 ± 4.3	16.5 ± 4.3
	Length between the T point-artery (mm)*	−4.2 ± 13.6	−2.5 ± 13.4
(A)	Subcutaneous thickness (mm)	5.9 ± 2.5	8.0 ± 2.2
	Depth from skin to artery (mm)	18.3 ± 4.0	18.8 ± 4.1
	Length between the A point-artery (mm)*	−2.5 ± 13.6	−3.7 ± 15.8

The T point is the injection site based on the three-finger breadth method.

The A point is the injection site based on the axillary method.

*The length between the adjacent artery visible on ultrasound and the T or A point. The value is positive if the artery is anterior to the point and negative if the artery is posterior to the point.

The BMI and subcutaneous thickness at the T point were positively correlated in men (r = 0.75, p < 0.001) and women (r = 0.45, p < 0.001) (Table 2). The BMI and subcutaneous thickness at the A point were positively correlated in men (r = 0.81, p < 0.001) and women (r = 0.55, p < 0.001). The upper arm circumference and subcutaneous thickness at the A point were positively correlated in women (r = 0.58, p < 0.001).

**Table 2:** The summarize of correlation analysis and prediction equation of subcutaneous thickness.

**Men (n = 134)**			**Women (n = 111)**		
BMI and SCT at	T point	r = 0.75	BMI and SCT at	(T)	r = 0.45
	A point	r = 0.81		(A)	r = 0.55
UAC and SCT at	T point	r = 0.68	UAC and SCT at	(T)	r = 0.51
	A point	r = 0.73		(A)	r = 0.58

SCT: subcutaneous thickness.

T: The injection point performed by three-finger breadth method.

A: The injection point if the vaccination is performed by axillary method (See Fig. 1).

UAC: upper arm circumference.

The prediction equations for subcutaneous thickness at the A point are:

(1) 0.62×BMI-7.7mm(men)

(2) 0.658×BMI-5.5mm(women)

The R^2^ values of the prediction equations are 0.66 for equation (1) and 0.31 for equation (2) (Table 3).

**Table 3:** Prediction equation of subcutaneous thickness predicted from BMI and upper arm circumference.

**Men (n = 134)**			
Predicted SCT at	T point (mm)	(0.39 × BMI)-3.9	R^2^ = 0.56
	A point (mm)	(0.62 × BMI)-7.7	R^2^ = 0.66
	T point (mm)	(0.034 × UAC)-5.6	R^2^ = 0.46
	A point (mm)	(0.054 × UAC)-10.2	R^2^ = 0.53
**Women (n = 111)**			
Predicted SCT at	T point (mm)	(0.40 × BMI)-2.1	R^2^ = 0.20
	A point (mm)	(0.66 × BMI)-5.5	R^2^ = 0.31
	T point (mm)	(0.038 × UAC)-4.1	R^2^ = 0.26
	A point (mm)	(0.058 × UAC)-5.5	R^2^ = 0.34

SCT: subcutaneous thickness.

T: The injection point performed by three-finger breadth method.

A: The injection point if the vaccination is performed by axillary method (See Fig. 1).

UAC: upper arm circumference.

One participant required several minutes of compression hemostasis after vaccination. No participants had symptoms of SIRVA during the study.

## Discussion

In this study, equations to predict the appropriate depth of deltoid muscle injections based on BMI were determined. Our results indicate that the new deltoid muscle injection method proposed by Nakajima et al. [7] is safe and appropriate due to similar results to Nakajima et al. by a larger number of people.

BMI is a quick, useful, and intuitive predictor of fat mass percentage [10]. Several studies have proposed appropriate depths of needle insertion based on BMI [8], [11]. To our knowledge, this is the first study including data collected at a COVID-19 vaccination event that investigates vaccine administration methods. Cook et al. reported that BMI is correlated with subcutaneous thickness in elderly people (r = 0.71 in men, r = 0.79 in women), which is consistent with the results of this study for men. However, the results of this study regarding women differ from those of the previous study. This may be due to differences in age and BMI between the two study groups, as the female participants in the current study are younger and have a lower BMI than those in the previous study. We think this difference in results may also be related to menopause, and further investigation is needed. The correlation between BMI and subcutaneous thickness is not as strong among young, female participants, suggesting that there are variations in the subcutaneous thickness among participants with the same BMI in this population. This is an important finding, as a previous study described young, thin women as being at risk of SIRVA [12]. A previous review noted that 82.6% of suspected SIRVA occurs in women [13]. The results of the current study may be related to these SIRVA risk factors. Interestingly, the correlations between upper arm circumference and subcutaneous thickness at the T and A points were similar to the correlations of BMI and subcutaneous thickness in women participants in this study, suggesting that upper arm circumference can substitute BMI to predict appropriate depth of needle insertion in young women. Upper arm circumference is becoming a possible alternative to BMI, as it has been reported as useful in screening for over- and underweight individuals [14], [15]. As upper arm circumference can be measured easily, it is important to consider this method to predict the appropriate injection site and depth.

The results of this study suggest that the new deltoid muscle injection method proposed by Nakajima et al. [7] is safe. There are four typical muscle injection points on the shoulder: three finger breadths (5 cm) below the mid-acromion, a triangular injection site, the middle third of the deltoid muscle, and a mid-deltoid site. Cook et al. warned that injections into these sites may result in injuries to the subdeltoid, subacromial bursa, or the anterior branch of the axillary nerve [8]. Therefore, Nakajima et al. advocated injections at the position referred to as the A point in this study [7]. This method has been termed the axillary method. In the axillary method, intramuscular injection is performed at a lower position than the conventional method (just below the midportion of the mid-acromion lateral border at the level of the upper end of the anterior and posterior axillary line). In recent reports, the safest anatomic site was found to be 7–13 cm below the mid-acromion [2]. However, the development of the axillary method was based on a limited number of volunteers (15 men and 15 women). The current study is unique in that it includes significantly more volunteers and includes prediction equations for subcutaneous thickness with respect to the axillary method. When 8 mm is added to the prediction equation, 97.8% of men and 96.4% of women meet the criteria for a puncture at least 5 mm from the fascia, which is considered appropriate for an intramuscular injection [11]. Due to deeper needle insertions with a higher frequency of inserting the needle beyond the adjacent arterial depth, 8 mm should be added to the prediction equation to determine the appropriate insertion depth. Additional examination that supports this discussion is summarized in Supplementary File 1.

The r-value for the A point is higher than that for the T point, regardless of the participant’s sex, in this study. We think these results do not indicate that the conventional three-finger breadth method is unsafe. No participant in this study developed SIRVA, although one required several minutes of compression hemostasis. The Health Service Center of Hirosaki University received no calls reporting shoulder dysfunction from the vaccinated individuals, including the 245 participants of this study. These data suggest that the three-finger breadth method is not dangerous when performed by a trained medical practitioner. The reports of SIRVA associated with the COVID-19 vaccine are increasing [3], [4], [5], [6], though this complication is not unique to the COVID-19 vaccine and is mostly caused by inappropriate injection techniques. A better understanding of shoulder anatomy and the establishment of an appropriate injection method based on that understanding may help prevent SIRVA.

This study has several limitations. First, it included predominantly young people. The study by Cook et al. may be considered more representative of the population [8]. Second, only one woman with a BMI > 25 was included in this study, suggesting that the prediction equation is less accurate for obese women. In contrast, this is also a strength of this study, as the correlation between BMI and subcutaneous thickness is stronger in men than in women despite the limited data that did not include obese women. This result may reflect the wide variation in the shoulder anatomy of young women. Third, although echographic data were obtained in this study, the clinical safety of the axillary method was not examined as all vaccines were administered using the three-finger breadth method.

## Conclusion

In conclusion, BMI and subcutaneous thickness in the shoulder are correlated in the young population. However, the subcutaneous tissue thickness varies more in women than in men. This study presents prediction equations for the appropriate insertion depth of intramuscular injections based on BMI for the three-finger breadth and axillary methods. Both the BMI and upper arm circumference can be used to predict the appropriate depth of intramuscular injections in women.

## Institutional Review Board Statement

The institutional board of Hirosaki University approved this study on January 21, 2022 (approval number 2021-140). The study was conducted in accordance with the Declaration of Helsinki.

## Informed Consent Statement

Written informed consent was obtained from all participants.

## Data Availability Statement

The data presented in this study are available in this article.

## Funding

This work was supported by the Public Foundation of Vaccination Research Center.

## CRediT authorship contribution statement

**Ryosuke Kowatari:** Funding acquisition, Writing – review & editing, Writing – original draft, Investigation, Validation, Methodology, Conceptualization. **Hanae Sasaki:** Supervision, Writing – original draft, Resources, Investigation, Validation. **Kenyu Murata:** Data curation, Resources, Formal analysis. **Ken Sato:** Formal analysis. **Koichi Sagawa:** Formal analysis. **Masako Kudo:** Data curation, Resources. **Masahito Minakawa:** Project administration, Formal analysis.

## Declaration of Competing Interest

The authors declare no financial interests.

## Data Availability

Data will be made available on request.
