# Supplementary material for: Establishment of prediction equations for subcutaneous tissue thickness in two representative intramuscular deltoid injections
Source: Vaccine X. 2023 May 22;14:100316. doi: 10.1016/j.jvacx.2023.100316 (PMC10239006; doi:10.1016/j.jvacx.2023.100316)
Supplement: Supplementary Data 1 [file mmc1.docx]

**Supplementary Table 1**: *Percentages of insertion depths >5 mm deeper than the fascia and percentages of insertion depths deeper than the proximal artery for the volunteers*

| **Men (n=134)** | **pSCT +5 mm** | **+6 mm** | **+7 mm** | **+ 8mm** | **+9 mm** | **+10 mm** |
| --- | --- | --- | --- | --- | --- | --- |
| 5 mm deeper than fascia | 56.7% | 76.1% | 89.6% | 97.8% | 98.5% | 100% |
| Insertion depth deeper than adjacent artery | 1.5% | 1.5% | 5.2% | 13.4% | 20.9% | 31.3% |
| **Women (n=111)** |  |  |  |  |  |  |
| 5 mm deeper than fascia | 50.5% | 72.1% | 89.2% | 96.3% | 96.3% | 99.1% |
| Insertion depth deeper than adjacent artery | 9.9% | 12.6% | 21.6% | 24.3% | 31.5% | 41.4% |

pSCT: predicted subcutaneous thickness (see Table 3).
